# The Cellular and Molecular Effects of Fetoscopic Endoluminal Tracheal Occlusion in Congenital Diaphragmatic Hernia

**DOI:** 10.3389/fped.2022.925106

**Published:** 2022-07-05

**Authors:** Oluyinka O. Olutoye II, Walker D. Short, Jamie Gilley, J. D. Hammond II, Michael A. Belfort, Timothy C. Lee, Alice King, Jimmy Espinoza, Luc Joyeux, Krithika Lingappan, Jason P. Gleghorn, Sundeep G. Keswani

**Affiliations:** ^1^Division of Pediatric Surgery, Department of Surgery, Texas Children's Hospital, Houston, TX, United States; ^2^Michael E. DeBakey Department of Surgery, Baylor College of Medicine, Houston, TX, United States; ^3^Division of Neonatology, Department of Pediatrics, Texas Children's Hospital, Houston, TX, United States; ^4^Texas Children's Fetal Center, Baylor College of Medicine, Houston, TX, United States; ^5^Department of Obstetrics and Gynecology, Baylor College of Medicine, Houston, TX, United States; ^6^Division of Neonatology, Department of Pediatrics, Children's Hospital of Philadelphia, Philadelphia, PA, United States; ^7^Department of Biomedical Engineering, University of Delaware, Newark, DE, United States

**Keywords:** congenital diaphragmatic hernia (CDH), tracheal occlusion (TO), FETO, pulmonary hypoplasia, pulmonary hypertension, pulmonary development, cellular and molecular factors

## Abstract

Congenital diaphragmatic hernia (CDH) is a complex disease associated with pulmonary hypoplasia and pulmonary hypertension. Great strides have been made in our ability to care for CDH patients, specifically in the prenatal improvement of lung volume and morphology with fetoscopic endoluminal tracheal occlusion (FETO). While the anatomic effects of FETO have been described in-depth, the changes it induces at the cellular and molecular level remain a budding area of CDH research. This review will delve into the cellular and molecular effects of FETO in the developing lung, emphasize areas in which further research may improve our understanding of CDH, and highlight opportunities to optimize the FETO procedure for improved postnatal outcomes.

## Introduction

Congenital Diaphragmatic Hernia (CDH) is a congenital anomaly characterized by a developmental diaphragmatic defect leading to herniation of abdominal contents into the thorax. This anatomic defect results in compression of the developing lungs by the herniated organs, leading to pulmonary hypoplasia and aberrations in pulmonary vascular development, which clinically manifests as pulmonary hypertension (CDH-PH). Airway pathology in CDH consists of decreased branching morphogenesis, which is associated with a reduced number of alveoli and decreased airway compliance at birth that manifests as hypoxic respiratory failure postnatally (1, 2). Morphological changes in the pulmonary arteries of patients with CDH include increased arterial wall thickness and pathologic remodeling of the extracellular matrix (3, 4). Collectively, these changes contribute to the increased pulmonary vascular resistance which is a critical component of CDH-PH pathophysiology (5) and is variably responsive to traditional vasodilatory therapies (6). Thus, the combination of immature airways and morphological changes in the pulmonary vessels in infants with CDH result in decreased gas exchange and hypoxemic respiratory failure: the crux of mortality in CDH.

Morbidity and mortality in CDH are driven by reversible and irreversible mechanisms that can limit the efficacy of postnatal therapy. CDH-PH represents a reversible or treatable component of the disease and is the target of most frontline vasodilatory postnatal therapies in CDH (7). Lung hypoplasia, however, is an irreversible or fixed component of the disease, and its prenatal assessment is often used as a predictor of disease morbidity and mortality (8). There are currently no well-described, postnatal methods of increasing lung size in infants with CDH apart from allowing the normal postnatal terminal differentiation of lung parenchyma.

**Figure 1:**
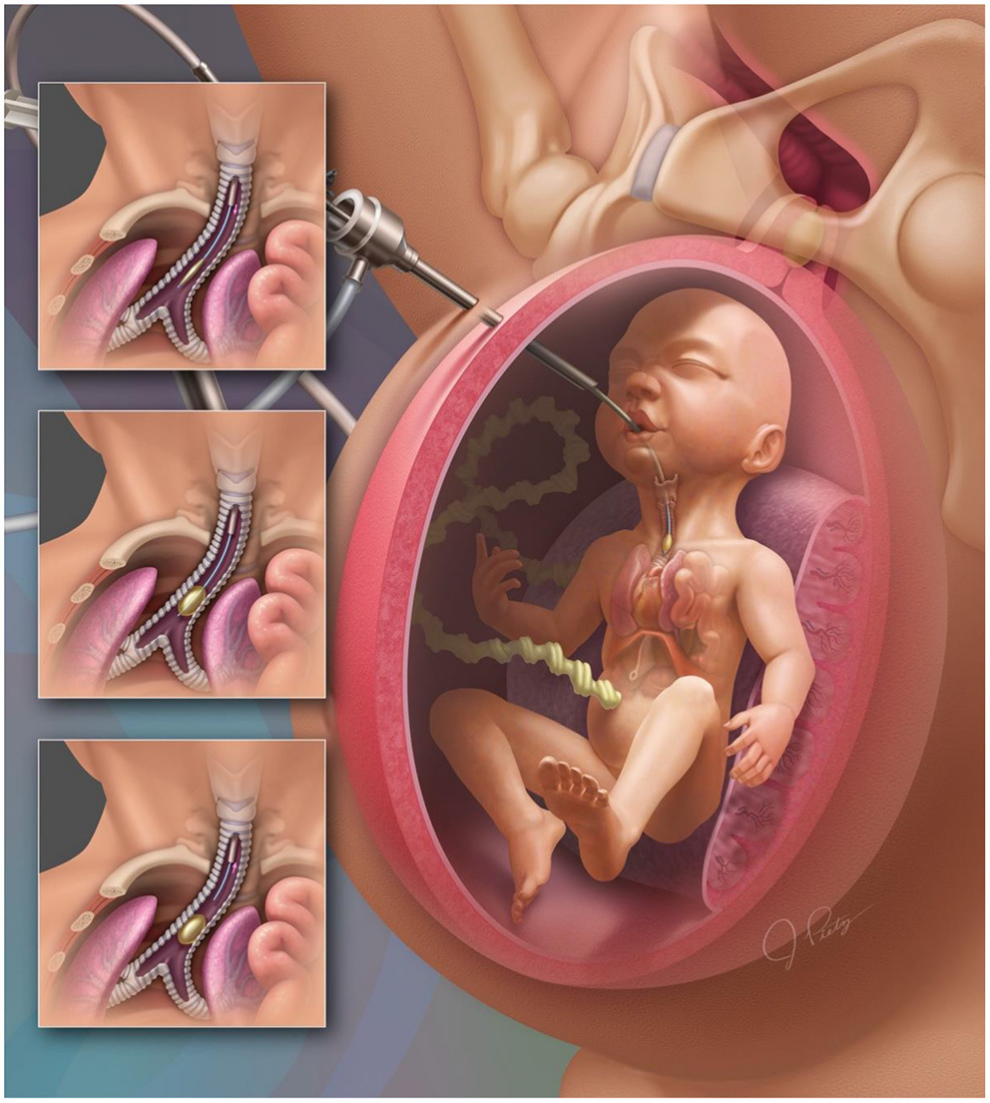
Fetoscopic Endoluminal Tracheal Occlusion with balloon inflation.

One strategy to address this specific component of CDH lung maldevelopment is to occlude the trachea in the fetal period, with a goal of increasing functional lung volume prior to delivery in a procedure named Fetoscopic Endoluminal Tracheal Occlusion (FETO) (9) (Figure 1). This innovative treatment has been proven safe, and previous studies including a recent randomized clinical trial, demonstrated that it was effective in increasing functional lung volume and improving postnatal PH outcomes for infants with more severe prenatal markers of CDH (10–13). This work aims to describe the cellular and molecular effects of FETO on the developing lungs of CDH patients and provide clinicians with a fundamental understanding of the effects of FETO. It begins with a brief history of tracheal occlusion in CDH and discusses what is known concerning the effects of tracheal occlusion on the fetal pulmonary airways, pulmonary vasculature, and the pulmonary cellular and molecular microenvironment. Additionally, this literature review will aid researchers in identifying areas where further research can increase our understanding of CDH and leverage opportunities to optimize the FETO procedure for improved postnatal outcomes.

## Fluid Pressure in Pulmonary Development and the Inception of Tracheal Occlusion

Biomechanical forces on the fetal lung play an integral role in fetal lung development and are dependent on fetal lung fluid and fetal breathing movements (14, 15). Fetal lung fluid is maintained in the lung by restricted egress at the larynx, creating a relatively pressurized system that is necessary for normal bronchial branching (16, 17). The transmural pressure in this system does not remain constant, however. The system experiences cyclic changes in pressure and fluid flow due to peristaltic movements of smooth muscle surrounding the airways, which, along with fetal breathing movements, propagate pressure waves to the distal tips of the airways (18). This cellular mechanotransduction pathway contributes to airway epithelial tree growth (19, 20), alveolar cell differentiation (16), and thus, surfactant production. As gestation progresses, this fluid leaves the lung via the larynx and either enters the amniotic fluid or is swallowed by the fetus (21, 22).

Defects in lung maturation occur when the fluid forces of pulmonary development are disrupted. In conditions such as congenital high airway obstruction syndrome, pulmonary hyperplasia is noted secondary to increased intrapulmonary pressure (23). Conversely, pathologic conditions, such as oligohydramnios or fetal neuromuscular diseases that interfere with fetal breathing and decrease fetal lung distension, lead to pulmonary hypoplasia (14, 24). Given the importance of prenatal airway fluid pressure in proper lung development (14), a logical theory emerged that iatrogenic tracheal occlusion would promote lung growth in patients with CDH (25).

*In utero* tracheal occlusion and its effects on the developing fetal lung have been examined in-depth as far back as 1948, when Jost and Policard decapitated fetal rabbits *in utero*, leading to tracheal occlusion, and found that alveoli were larger on histology at delivery (26). In 1965, Carmel et al. further studied lung development in the fetal rabbit (27); they found that lung weight and volume increases following tracheal ligation, signifying that the leporine lung produced its own fluid that aided in its growth, independent of amniotic fluid entering the lung. Similarly, in fetal lambs, tracheal ligation was found to increase both lung volume and alveolar development as compared to lambs whose airways were drained of fluid *in utero* (28). These same effects on lung growth patterns were seen in human fetuses with tracheal obstructions due to congenital laryngeal atresia and tracheal agenesis (29). With postnatal lung hypoplasia being a major, irreversible component of morbidity and mortality in CDH, these findings stimulated further investigation into how occluding the trachea could potentially augment lung development in CDH.

In 1993, Wilson et al. demonstrated that silk tie tracheal ligation of fetal sheep status post fetal nephrectomy alleviated the expected pulmonary hypoplasia associated with fetal nephrectomy, suggesting a potential application to fetuses with CDH (30). They then went on to perform *in utero* tracheal ligation in a CDH sheep model and found significantly increased alveolar surface area, alveolar number, lung to body-weight ratio, and lung compliance, when compared to the controls without tracheal ligation (31). In 1994, Harrison and colleagues also demonstrated that, in a fetal lamb model of CDH, *in utero* ligation of the trachea led to growth of the previously hypoplastic lungs, return of abdominal viscera to the abdomen, and significantly increased postnatal oxygenation and ventilation of lungs (32). Furthermore, they demonstrated that it was possible to occlude the trachea *in utero* with positive outcomes using a P.L.U.G. (Plug the Lung Until it Grows) method, solidifying *in utero* tracheal occlusion as a promising therapy for human fetuses with CDH. Two methods to occlude the trachea were tested by the team: tracheal placement of a gelatin encapsulated foam insert or external tracheal clips in a fetal lamb model of CDH during an open surgical procedure. Both groups of fetuses displayed improvements in pulmonary function at birth (33). The team later adapted their technique from P.L.U.G. to a “Fetendo-PLUG” by endoscopically placing foam inserts into the fetal lamb's trachea with a bronchoscope and tracheal clips via anterior neck incisions. These studies showed that it is possible to successfully occlude the trachea internally and externally via small incisions and endoscopy, without the need to make a large incision in the uterus (34, 35).

Prior to the introduction of the PLUG technique, attempts had been made to repair the anatomic defect in the diaphragm *in utero* in human CDH patients. While *in utero* repair had been proven technically feasible in patients without liver herniation, these patients received no additional benefits to their outcomes vs. patients receiving postnatal repair (36). Furthermore, when repair was attempted in patients with liver herniation, it led to high rates of prenatal death due to kinking of the umbilical vein (37). By contrast, the PLUG procedures relied on occlusion of the trachea, which could be completed fetoscopically and did not involve surgical manipulation near the umbilical cord. Based on promising preclinical evidence in lamb models (34), the procedures were translated to initial feasibility trials in human fetuses with CDH that were predicted to have a poor prognosis based on the degree of liver herniation, early diagnosis (before 25 weeks gestation), and low lung-to-head ratio (LHR) (9). In a study of fetuses with left CDH who had liver herniation and a low LHR, both the fetuses and their mothers were found to have improved outcomes following the Fetendo-PLUG procedures for temporary tracheal occlusion as compared to open tracheal occlusion (9). In 2000, a new method of fetoscopic endoluminal tracheal occlusion (FETO) was introduced that involved balloon tracheal occlusion. Testing in a fetal lamb model showed that the balloon was able to occlude the trachea more accurately and cause less tracheal damage, since balloon inflation could be tailored to the trachea size (38). This method of occlusion has now been trialed in humans and is routinely performed at high volume centers in CDH patients with moderate to severe lung hypoplasia (11, 12).

## Cellular and Molecular Effects of Tracheal Occlusion

### Pulmonary Airway System

**Figure 2:**
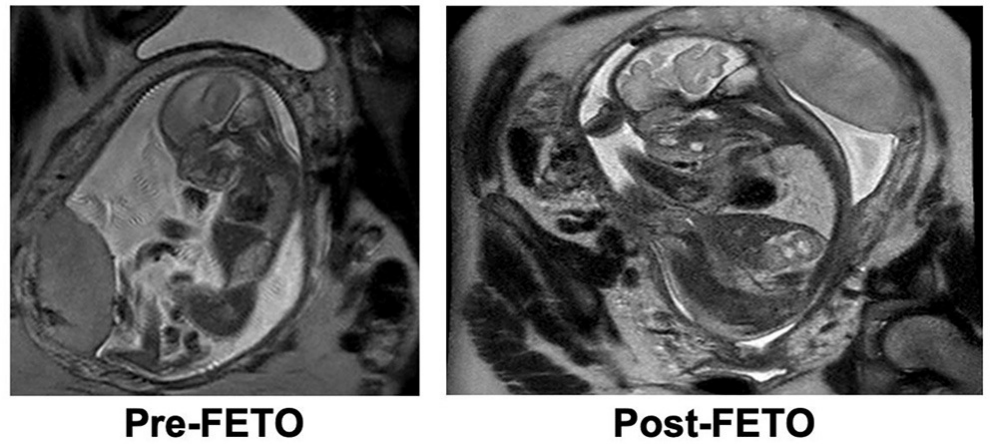
Increase in observed-to-expected lung volume following prenatal intervention with Fetoscopic Endoluminal Tracheal Occlusion.

Tracheal occlusion has been shown to increase lung dry weight, airway branching morphogenesis, total air exchanging parenchyma, and overall lung maturation (27, 39, 40). Temporary tracheal occlusion in fetuses with CDH increases the degree of lung parenchyma involved in gas exchange to values comparable with non-CDH fetuses, as shown in a fetal lamb model (40). Following FETO intervention in human CDH patients, an increase in the observed-to-expected fetal lung size can be seen as early as 1 week, and lung hypoplasia is attenuated at birth (33) (Figure 2).

In a 1996 study by Glick et al., researchers performed tracheal ligation in a lamb model of CDH (41). From 110 days gestation until term, surfactant production by alveolar type II pneumocytes initially decreased after tracheal ligation. Lung volume and gas exchange increased in the ligated group vs. controls; however, surfactant presence, measured by rate of tritiated choline incorporation into phosphatidylcholine, was lower. The authors hypothesized that this was due to improper type II alveolar pneumocyte maturation because of the lack of rhythmic stretch from fetal respiration with tracheal occlusion. Prior studies showed that cyclic strain applied to pulmonary type II alveolar epithelial cells increased surfactant phospholipid synthesis *in vitro* (42) and lack of fetal breathing movements in fetal lambs inhibited type II alveolar pneumocyte maturation (43). It was shown that abolition of fetal respiratory movements by transection of phrenic nerves, coupled with the increased fluid in the lungs, caused overstretching of type II alveolar pneumocytes and dysfunctional surfactant production. This led to consideration of a graded tracheal occlusion device with a “pop-off valve” that could then restore tracheal continuity (41). Other studies have examined timing of tracheal occlusion and the benefits of temporary occlusion vs. long-term occlusion in lamb and rabbit models. These studies found that occlusion for longer periods of time and at points earlier in gestation led to lung growth but also pulmonary overstretching, which significantly limited type II alveolar pneumocyte function and contributed to their depletion (40, 44–46). Studies continue to show that tracheal occlusion restores type I alveolar pneumocyte differentiation but that pulmonary overstretching may adversely affect surfactant production in type II alveolar pneumocytes (47).

Papadakis et al. explored the idea that prolonged tracheal obstruction in a fetal lamb CDH model led to both lung growth, decline type II pneumocytes population, and surfactant deficiency. They compared fetal lamb CDH models with tracheal ligation to those with temporary occlusion at 108 days gestation. In the temporary occlusion model, the occlusion was removed after 14 days. They found that the lambs with temporary tracheal occlusion had a larger total parenchymal lung volume than CDH lambs with no occlusion. In fact, the lung volumes for these treated CDH lambs approached the volume of control lungs, although they remained smaller on average. This study, along with fetal sheep studies by other groups demonstrated significant increases in lung weight and volume following temporary occlusion (40, 48, 49). Taken together the aforementioned studies provided the rationale for temporary tracheal occlusion, as it is effective in increasing the total lung volume, but also diminishes negative effects prolonged tracheal occlusion has on surfactant production by type II pneumocytes.

**Figure 3:**
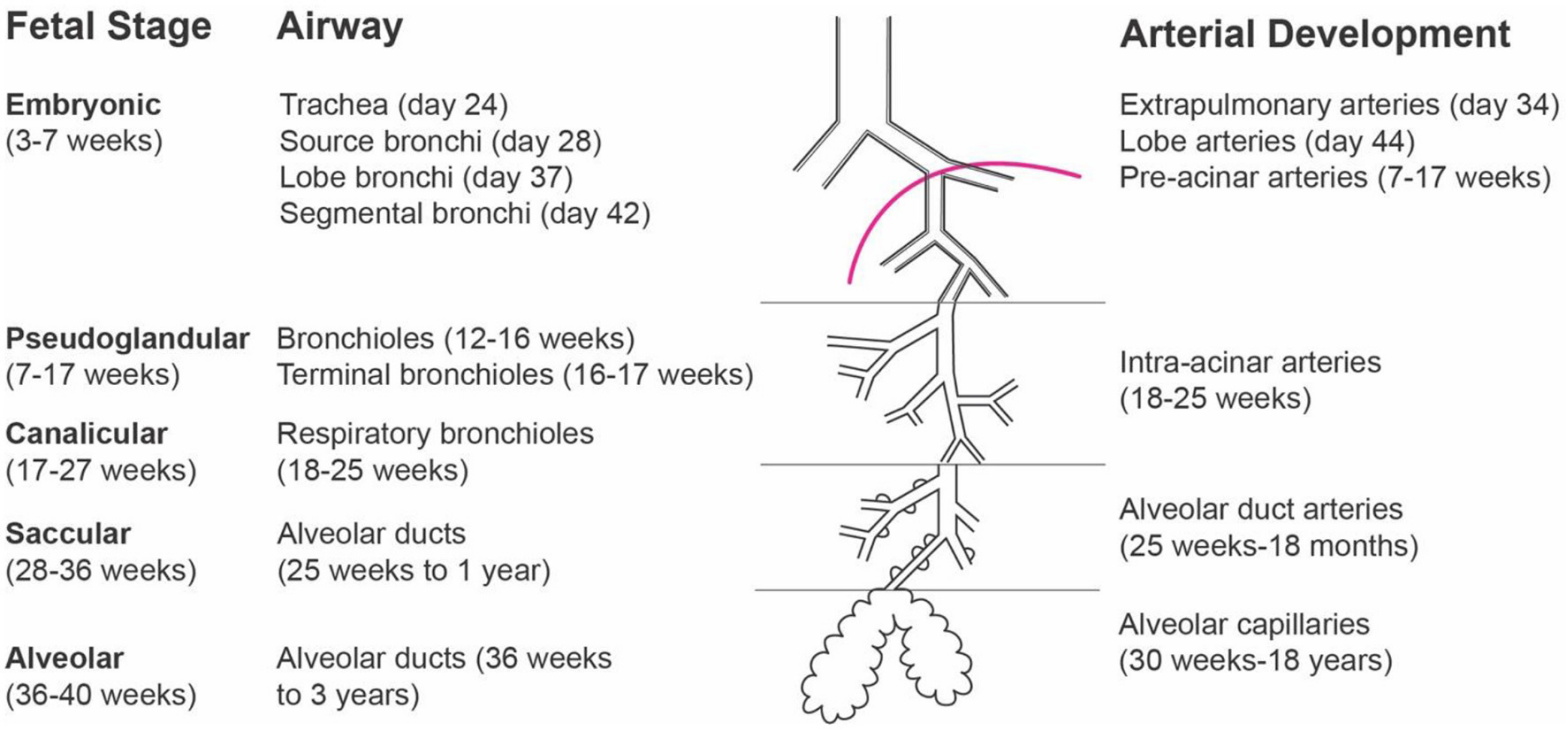
Stages of fetal lung development.

In addition to the discovery of unintended consequences of long-term occlusion, there has also been intense discussion around when occlusion should be performed. A study by Liao and colleagues explored the question of whether lung growth is affected by the gestational timing of tracheal occlusion. They demonstrated that fetal lambs that underwent tracheal occlusion around 122 days, rather than the usual 108 days, underwent a period of rapid lung growth after occlusion. Ultimately, though, the authors failed to detect a difference in lung size between the 108-day group and the 122-day (late occlusion) group by the end of gestation. However, they did measure a greater air-space fraction and improved preservation of the type II alveolar pneumocyte population in the late occlusion group (46), suggesting that occlusion later in gestation may be more beneficial than long-term occlusion. Subsequently, these findings have been reproduced in a rabbit model of CDH (45) and validated in humans, where FETO has been shown to increase total fetal lung volumes, with lung growth in the first week after FETO being a positive predictor of improved postnatal outcomes (50, 51). We now know that tracheal occlusion better affects lung growth during the canalicular stage of lung development (16–26 weeks gestation in humans), more so than the saccular stage (24 weeks to term gestation in humans), as gestational age at the time of tracheal occlusion has been demonstrated to be an independent predictor of fetal lung volume (52, 53) (Figure 3). Thus, negative effects on type II alveolar pneumocytes are attenuated when tracheal occlusion is done later in gestation and when there is an *in utero* interval between cessation of occlusion and birth (46).

Both the disease (CDH) and the intervention (FETO) affect the function of type II alveolar pneumocytes, and production of surfactant by type II alveolar epithelial cells is a key factor mediating the outcomes in CDH. However, the question of whether the developing CDH lungs are surfactant deficient or rather contain proportionate amount of surfactant for the lung volume has been an area of controversy. It is known that surfactant level is a metric of fetal lung maturity (54, 55) and that its production is regulated by neuroendocrine factors (56). Surfactant is composed of 90% lipids and phospholipids, especially phosphatidylcholine, which dominates the lipid fraction of surfactant (57). Surfactant deficiency was initially thought to be a major contributor to the poor outcomes in CDH, given the low amount of phospholipids present in amniotic and tracheal fluid. In their 1992 study, Glick et al. found that total phospholipids and percent phosphatidylcholine were reduced in lavage samples of fetal lung tracheal fluid obtained from lambs with experimental CDH, suggesting a reduced amount of surfactant was produced (58). However, in 1994, Sullivan et al. studied human amniotic fluid samples and found no difference in the lecithin to sphingomyelin ratio or phosphatidylglycerol levels in CDH vs. control samples (59). This suggested that there was no quantitative decrease in the amount of surfactant in fetuses with CDH and that the perceived surfactant decrease in CDH lungs was the result of normal surfactant production but in a hypoplastic lung. However, these normal findings were questioned due to the methods employed. One critique suggested that the pooled amniotic fluid samples collected included contents of both lungs and that surfactant production by the more mature contralateral lung may mask the immature ipsilateral lung.

To address this question more definitively, others went on to investigate surfactant production in human CDH fetuses and CDH lamb models. They found that concentrations of disaturated phosphatidylcholine and surfactant proteins were similar, with both components exhibiting a similar pattern of increase throughout gestation in human fetuses with CDH and in control fetuses with non-pulmonary diseases (60). Keratinocyte growth factor (KGF) and neuregulin, two glucocorticoid-regulated mediators of lung epithelial cell maturation, were also measured. KGF decreased with time in controls but remained unchanged in CDH human fetuses, while neuregulin increased late in pregnancy in both CDH human fetuses and controls. These results contrasted with results obtained from a lamb model of CDH, where levels of both KGF and neuregulin were lower in CDH lambs than controls. However, treating the lamb model with FETO partially restored KGF expression and fully restored neuregulin expression (61). Thus, in human CDH patients and in a FETO-treated lamb CDH model, the lung maturation factors did not show a distinct downward trend, supporting the notion that CDH lungs are not deficient in maturation factors that would promote surfactant production and are thus not surfactant deficient. Mimmi et al. went on to examine the effect of FETO on surfactant production in the fetal CDH lamb model. They found that while CDH induction decreased phosphatidylcholine levels compared to healthy lambs, temporary tracheal occlusion returned expression to a physiologic level (62).

Overall, the evidence for surfactant deficiency in CDH is not overwhelming, and surfactant continues to be used as a therapy for CDH patients with ventilator dependence in some institutions. Altered surfactant production profile may play a role in humans with CDH, but overall production is not decreased when compared to age-matched controls without CDH and surfactant production in the hypoplastic lung of a CDH fetus increases at a similar rate to the lungs of normal fetuses (61). Temporary tracheal occlusion has been shown to positively impact the expression of key factors in lung maturation in the fetal lamb CDH model. Further studies are needed to characterize the effect of FETO on type II pneumocytes, surfactant levels, and the expression of molecular factors involved in lung maturation in the human fetus with CDH.

### Pulmonary Vasculature

#### Physiologic Effects of FETO on the Pulmonary Vasculature

The development of a normal lung parenchyma and pulmonary vasculature are tightly linked. As such, the perturbations seen in lung development in infants with CDH are known to cause associated abnormalities in pulmonary vascular development. Hallmarks of pulmonary vascular maldevelopment in CDH include a hypoplastic vascular bed that often demonstrates inadequate response to pulmonary vasodilators (63, 64). In rabbit models of CDH, tracheal occlusion is associated with increased lung perfusion as measured by fractional moving blood volume (FMBV) and pulsatility index (65, 66). These features have been validated in humans with CDH, where FETO was associated with a 30% increase in FMBV, which the authors also put forward as an additional prognostic indicator in humans with CDH (67). This trend has also been noted clinically in human subjects, as FETO patients have lower resistance at the first branch of the pulmonary arteries than their untreated counterparts (68). Furthermore, neonates with moderate CDH that have undergone FETO have a higher likelihood of resolution of PH by discharge than those that did not undergo FETO (13). In human patients with severe CDH, FETO leads to a significantly higher rate of resolution of PH by 1 year after birth than in infants who did not undergo FETO (69). These results suggest that tracheal occlusion alters pulmonary vessels to create improvements in gas exchange and perfusion.

In human CDH-PH, extensive muscularization of pulmonary vessels is evident as early as 19 weeks of gestation, and contractile pulmonary vascular smooth muscle cells are more abundant earlier in gestation in patients with CDH than in controls (70). In rat, rabbit, and lamb models of CDH that underwent temporary *in utero* tracheal occlusion, the thickness of the pulmonary artery medial layer was significantly reduced, with values comparable to or less than those that did not undergo tracheal occlusion (31, 66, 71, 72). Pulmonary artery adventitial thickness was also reduced with tracheal occlusion in a rat model of CDH (73, 74). However, these findings were not reproducible in humans according to Danzer et al., who found that open prenatal tracheal occlusion did not change the medial thickening in pulmonary arteries <140 μm (75). However, these parameters were measured in patients that received tracheal occlusion without release, thus there is a need for new studies to examine these changes in the context of temporary tracheal occlusion.

#### Molecular Effects of FETO on Pulmonary Vasculature

Nitric oxide is thought to be the primary endothelial vasodilator in the pulmonary vasculature and is produced by three isoforms of nitric oxide synthase (NOS): neuronal NOS, inducible NOS (iNOS), and endothelial NOS (eNOS) (76). iNOS expression is increased in the lungs of the nitrofen-induced rat model of CDH compared to wild type, but once treated with ventilation or *in utero* indefinite tracheal occlusion, lung expression of iNOS decreases (74). Studies examining expression of iNOS have not been performed with temporary tracheal occlusion. Similar to iNOS, eNOS expression is known to be higher in the nitrofen rat CDH model than controls (77) and decreases with tracheal occlusion. Furthermore, eNOS could play a role in regulating the production of vascular endothelial growth factor (VEGF), which is known be increased in rat models of CDH that undergo tracheal occlusion (78, 79).

VEGF is an angiogenic factor secreted by type II pneumocytes that induces endothelial cell growth, proliferation, and angiogenesis in the lungs. VEGF is significantly lower in nitrofen rat models of CDH than in controls (79), an effect that can be rescued by tracheal occlusion as evidenced by increases in expression of VEGF-A mRNA and VEGF-A protein. Tracheal occlusion was also found to accelerate the maturation patterns of VEGF-A mRNA expression that are present in normal fetuses (80). Expression of VEGF-A mRNA in pulmonary vascular smooth muscle cells is known to be increased by cyclic stretch of the lungs (80, 81), thus it is thought that distention of the lungs caused by tracheal occlusion leads to this increased expression of VEGF-A (80). The previously stated studies, however, did not examine VEGF expression in temporary tracheal occlusion, such as the FETO procedure in humans. Further studies examining VEGF levels in animal models or infants with CDH that have undergone temporary tracheal occlusion will further delineate the role of VEGF-A in CDH.

In direct opposition to the vasodilatory effects of NOS, endothelin is a peptide that triggers vasoconstriction in vascular endothelial cells (82). Endothelin and endothelin receptor protein levels are lower in CDH lamb models that did not undergo tracheal occlusion than those that underwent tracheal occlusion. Still, endothelin protein expression is decreased in CDH with or without tracheal occlusion compared to normal controls, although expression of its receptor is increased in CDH with or without tracheal occlusion compared to normal controls (83). This demonstrates that while the endothelin system plays a role in PH, its role in mediating PH via tracheal occlusion is limited. Further research is needed regarding the role of endothelin in PH and, thus, its potential as a therapeutic target for interventions in the management of CDH-PH.

## Extracellular Vesicles and miRNA in CDH

More recently, extracellular vesicles (EVs) and their micro-RNA (miRNA) content in amniotic fluid and tracheal aspirates have been studied for their potential prognostic value in CDH. They have been identified for both positive and negative effects on the developing lung, and further study may yield advancements in lung disease and pulmonary hypertension therapeutics (84–86). While FETO has been shown to improve lung growth, proteomic analysis of amniotic and tracheal fluid following FETO have also shown that FETO substantially alters the lung at the cellular level (87) in ways that are still being elucidated. The discussion below explores recent studies on the role of miRNAs in CDH and FETO that may have relevance for future diagnostics and therapeutics.

One such study displayed that, while mir-200 expression is increased in CDH patients in comparison to healthy controls, the miR-200 family of miRNAs are enriched in amniotic fluid at the time of FETO balloon removal in CDH survivors compared to non-survivors and enriched in tracheal fluid of CDH patients that exhibited significantly lung volume increase with FETO compared to those who did not respond to FETO. Downstream effects of TGF- β induced signaling pathways are initiated via SMAD phosphorylation, and the TGF- β signaling transduction pathway has been identified as a major target of the mir-200 family (88, 89). In the absence of exogenous TGF- β, human bronchial epithelial cells were found to exhibit low SMAD-dependent gene expression, despite addition of mir-200b mimics. However, inhibition of mir-200b significantly increased SMAD-induced gene expression, suggesting that steady-state levels of mir-200b may play a role in basal TGF- β-induced signaling (90). This understanding of the role of the mir-200 family in TGF- β induced signaling, along with previous studies showing that TGF- β enhances mir-200b expression as part of a negative feedback loop (91), is increased by FETO in the hypoplastic lungs of CDH animal models (92), and may be increased by mechanical stretch of diseased lungs (93–95), gives further insight into the cellular and molecular effects of FETO. The increase of mir-200 seen at FETO release may be both due to the baseline increased expression of TGF- β in the hypoplastic lung and the increased of TGF- β secondary the mechanical stretch that FETO induces.

Other studies, however, have shown that increased intrapulmonary release of miRNA-rich, endothelial-derived microvesicles in response to chemical and mechanical lung injuries trigger alveolar macrophage-regulated inflammatory responses and cytokine release (96–98). Studies of human infants with CDH found that extracellular vesicle concentrations were higher in infants who underwent temporary tracheal occlusion and died postnatally, than in survivors (99). Interestingly, when the fetal amniotic and tracheal fluid of non-survivors were examined, a different profile was observed before and after the FETO procedure. Namely, mir-379-5p and mir-889-3p were both over-expressed in pre-FETO amniotic fluid, but mir-223-3p and mir-503-5p were over-expressed in the tracheal fluid at the time of FETO reversal, suggesting that these markers may indicate either disease progression or amelioration, respectively, depending on their balance in tracheal aspirates or amniotic fluid (99).

Both mir-223-3p and mir-503-5p were overexpressed in tracheal aspirates at FETO reversal in CDH FETO survivors, when compared to non-survivors. Thus, it is possible that these miRNAs are responsible for mediating or among the players involved in the regulation pathways that induce the overall positive effects of FETO on CDH outcomes, such as pulmonary hypertension (99). Supporting this notion, a study in a rat model of PH showed that increased expression of mir-223-3p was associated with reduced proliferation of pulmonary arterial smooth muscle cells and improved cardiovascular parameters (100). Mir-223-3p has also been shown to alleviate the progression of PH by suppressing expression of integrin β3 gene, a gene known to promote PH via aberrant pulmonary arterial smooth muscle cell proliferation when upregulated (100). Similarly, mir-503-5p was downregulated in rat models with PH (101), yet was overexpressed in fluid aspirates obtained *in utero* following FETO. Mir-503 is a key miRNA regulated by the apelin gene and downregulates the fibroblast growth factor-2 and fibroblast growth factor receptor-1 components of the endothelial fibroblast growth factor signaling network. Overexpression of mir-503-5p was shown to decrease proliferation of normal pulmonary artery endothelial cells (PAECs) and PH PAECs. Thus, FETO appears to upregulate miRNA networks that are associated with improved pulmonary arterial morphology and could be hypothesized to lead to improved outcomes in CDH. However, much work is needed to define both the roles of these networks, their specific roles in CDH-PH and FETO, and how the balance of their expression may be optimized to improve CDH outcomes.

Conversely, overexpression of mir-379-5p at FETO reversal promotes seemingly undesirable pathways in the developing lung. Mir-379-5p has been shown to directly target the 3'UTR region of the insulin-like growth factor 1 (IGF-1) gene, causing an inverse correlation between miR-379 and IGF-1 expression at both the mRNA and protein levels (102). IGF is an endocrine hormone that regulates cell proliferation and differentiation and mediates vasculogenesis in human lung development (103–105). As mir-379-5p increases, IGF-1 expression is decreased, theoretically decreasing the availability of IGF to promote cell proliferation, differentiation, and vasculogenesis. In this pathway, mir-379-5p overexpression at FETO reversal appears to be counterproductive to organized lung growth, however, this pathway is a relatively small portion of the larger picture of increased lung growth and improved post-natal outcomes observed with FETO intervention. Further study of the effect of FETO on mir-379-5p expression and pulmonary development pathways will help us better understand its role in fetal lung development. Besides the aformention miRNA targets, many other differentially expressed miRNAs and genes have been identified via transcriptome analysis in human CDH and a rabbit model of CDH (87, 106–108), some of which appear to serve a role in mediating the effects of FETO on the developing lung.

A step further from understanding the expression of microRNA following FETO, Zani and colleagues have gone on to understand the effect the addition of amniotic fluid derived stem cell extracellular vesicles (AFSC-EVs) to hypoplastic lungs. They found that, in the nitrofen rat model of CDH, addition of AFSC-EVs to fetal hypoplastic lungs rescues fetal lung underdevelopment by increasing the number of alveoli, decreasing the alveolar wall thickness, and promoting alveolar lipofibroblast maturation in nitrofen rat CDH models (109). Others have found that extracellular vesicles influence extracellular matrix remodeling in the pulmonary vasculature (110), and attenuate pulmonary endothelial cell PAE dysfunction (111) in the nitrofen rat model. Aside from FETO, there are currently no other evidence-based therapies for the *in utero* alleviation of pulmonary hypoplasia, pulmonary hypertension and their sequelae. This new knowledge, in addition to further understanding and characterization of the specific miRNA cargo of these extracellular vesicles, may lead to advancements in our prognostic abilities concerning outcomes in CDH and provides an opportunity to identify potential targets or deliverables for therapy as an evidence-based adjunct to FETO in the care of the CDH fetus.

## Summary

In summary, tracheal occlusion promotes lung growth and alveolarization in the hypoplastic and underdeveloped CDH lungs. However, studies have shown that temporary tracheal occlusion, as in FETO, is superior to indefinite tracheal occlusion. Temporary occlusion provides all the positive benefits of decreased pulmonary vascular thickness, decreased PH severity, and increased gas exchange while minimizing deleterious effects on type II alveolar pneumocytes.

**Figure 4:**
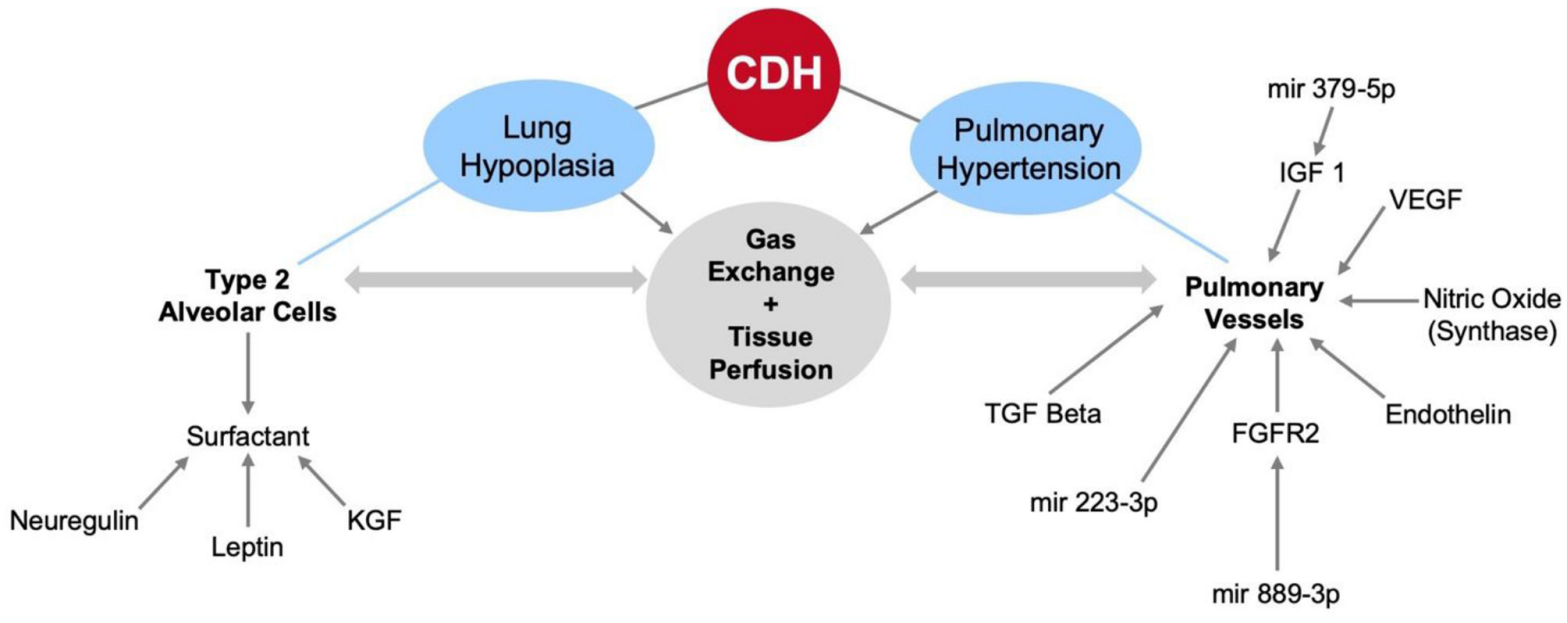
The cellular and molecular factors affecting pulmonary changes in fetoscopic endoluminal tracheal occlusion for CDH patients.

Further studies have shown that nitric oxide, VEGF, endothelin, neuregulin, and KGF mediate changes in pulmonary cellular and vascular development in the context of tracheal occlusion in CDH. Unfortunately, many of the studies discussed here were done in the setting of indefinite tracheal occlusion, thus additional studies in human and animal models are warranted to evaluate these factors before and following temporary tracheal occlusion. Such studies will give insight into the specific biological pathways involved in lung development that are perturbed by tracheal occlusion and possibly reveal new therapeutic targets to improve the efficacy of FETO. Novel research in extracellular vesicles and miRNA has also shown that tracheal occlusion regulates miRNA in the developing fetus, which can link differential gene expression to known factors mediating pulmonary development in CDH (Figure 4).

The introduction of FETO now allows clinicians to make inroads toward treating the previously irreversible aspect of CDH: lung hypoplasia. Surgeons continue to optimize FETO by developing new surgical techniques and devices that improve outcomes. One recent example is the development of a smart tracheal occlusion device that not only autoregulates lung fluid egression to optimize lung growth, but also eliminates the need for a second fetoscopic procedure to deflate the balloon (112). In the same vein, another group has developed a degradable hydrogel for tracheal occlusion that would also obviate the need for a second surgery (113).

However, much more research is needed to improve pulmonary hypertension—the reversible aspect of CDH. Understanding the cellular and molecular factors stimulated by FETO, specifically its effects on pulmonary vessel remodeling and gas exchange, may help us understand the biological pathways that can be targeted to reduce the morbidity and mortality of CDH- PH.

## Author Contributions

OOO and SGK conceptualized the manuscript content. OOO performed the literature review, drafted the manuscript, and reviewed the final version. OOO, WDS, JG, JH, MAB, TCL, AK, LJ, JE, KL, JPG, and SGK reviewed and edited the manuscript and approved the final product. All authors approved the final submission.

## Funding

This research was supported by the National Institutes of Health (T32HL139430 to OOO, R01HL144775 and R01146395 to KL, R01HL133163 to JPG, and R01HL140305 to SGK), and a Moorman Family gift to SGK.

## Conflict of Interest

The authors declare that the research was conducted in the absence of any commercial or financial relationships that could be construed as a potential conflict of interest.

## Publisher's Note

All claims expressed in this article are solely those of the authors and do not necessarily represent those of their affiliated organizations, or those of the publisher, the editors and the reviewers. Any product that may be evaluated in this article, or claim that may be made by its manufacturer, is not guaranteed or endorsed by the publisher.
